# Polyaromatic Cyclophanes Design and their Related Optical Properties

**DOI:** 10.1002/open.202400207

**Published:** 2024-12-04

**Authors:** Oumou Diallo, Jean‐Frédéric Audibert, Isabelle Leray, David Kreher, Guillaume H. V. Bertrand

**Affiliations:** ^1^ Université Paris-Saclay CEA, List F-91120 Palaiseau France; ^2^ Institut Lavoisier de Versailles (ILV) CNRS Université Paris-Saclay 45 avenue des Etats-Unis F-78035 Versailles France; ^3^ Université Paris-Saclay ENS Paris-Saclay CNRS Photophysique et Photochimie Supramoléculaires et Macromoléculaires 91190 Gif-sur-Yvette France

**Keywords:** polyaromatic cyclophane, [2,2] paracyclophane synthesis, Photophysics, chemical design of fluorescent molecules

## Abstract

In this article, we present several organic synthetic way to synthesize a family of five polyaromatic molecules based on a cyclophane core. Our strategies revolves around palado‐catalyzed substitution on a [2.2]paracyclophane (pCp) building block. Direct formation of a cyclophane was also employed for two molecules. The polyaromatic nature of the cyclophane library we synthetized made them good fluorophores candidate, we hence performed full photophysical characterization (Absorption, Emission, TCSPC) in different solvent as well as embed in polystyrene films. We evaluate how the cyclophane moiety influence their photo physical properties compared to their corresponding homologues without pCp core, demonstrating greater stoke shift and intramolecular exciplex behavior. The general behavior among cyclophanes was also compared and show solvent dependent properties as well as consistency of the photophysics between toluene and polystyrene matrix.

## Graphical abstract

This Study present the synthesis of polyaromatic cyclophane via different organic pathways. We performed a systematic photophysics study in solution (DCM and Toluene) as well as embed in polystyrene films. This allowed us to put forward the influence of the cyclophane ring on the photophysics properties; especially large stokes shift, intramolecular exciplex behavior and solvent dependent decay time.

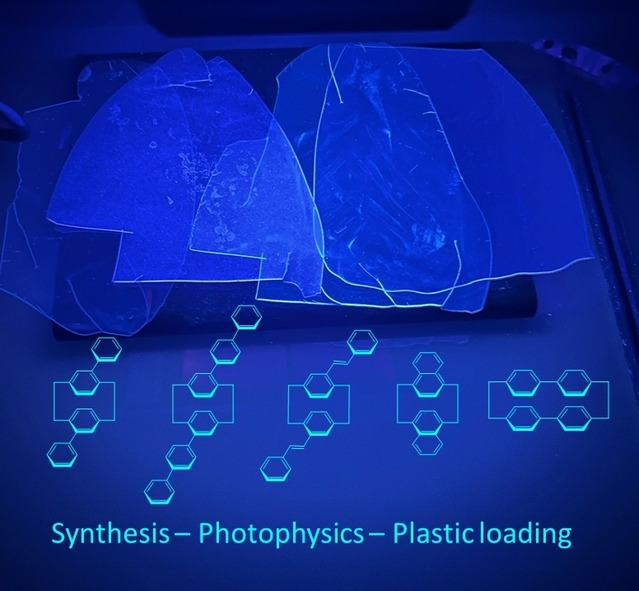

## Introduction

In 1949,^[1]^ Brown and Farthing uncovered [2.2]paracyclophane (pCp) as a by‐product during the low‐pressure pyrolysis polymerization of p‐xylene. Following this discovery, two years later, Cram and Steinberg^[2]^ introduced an alternative approach to synthesize pCp using intramolecular Wurtz reactions with metallic sodium on dibromide precursors. These pioneering studies marked the beginning of cyclophane chemistry, describing molecules containing both aromatic rings and aliphatic chains, allowing for a broad range of chemical modifications. Since that time, with the extensive structural diversity of cyclophanes, the methods used for their synthesis have been remarkably diversified.[^3^, ^4^, ^5^, ^6^]

The applications of molecules containing pCp are diverse and promising. For instance, they play a crucial role in the development of novel materials for water vapor capture, notably [2.2]paracyclophane‐based metal‐organic frameworks (MOFs), which exhibit enhanced performance in terms of capture capacity and recyclability.^[7]^ Furthermore, topologically chiral carbon nanocircles incorporating pCp units are under investigation for their potential applications in electronic devices and molecular machines.^[8]^ Recent research has also explored the synthesis of [2.2]paracyclophane‐based PRODAN analogues, wherein the naphthalene unit is replaced by a three‐dimensional framework, to probe the influence of through‐space conjugation on their photophysical properties.^[9]^ Notably, these studies have revealed the tunability of photophysical properties through interactions across space, suggesting prospects for designing customizable [2.2]paracyclophane‐based luminophores. Additionally, [2.2]paracyclophanes serve as fundamental building blocks in fields like supramolecular chemistry and guest‐host materials, leveraging their unique three‐dimensional structure and electronic properties to develop novel luminophores and artificial light‐harvesting systems.[^10^, ^11^]

Advancements in creating cyclophanes are intricately connected to the evolution of synthetic techniques for closing and contracting rings. Initially, studies were aimed at creating heterocycles capable of planar chirality or long‐range electronic communication, two features that heterocyclic compounds with the pCp substructure can offer. Analysis of its crystal structure by Cram and al. in 1971 revealed distinct behaviour in X‐ray measurements.^[12]^ The benzene rings, referred to as “decks,” exhibited a puckered conformation instead of being flat, with carbon atoms in the methylene bridges angled at 12.6° (boat conformation). This arrangement minimizes tensions associated with bond angles. The distance between the benzene cores was 3.09 Å, less than the typical π‐stacking distance (3.4 Å in graphite), indicating a robust overlap between the aromatic systems. Additionally, the C−C bond length of the methylene slightly extended to 1.55 Å, confirming molecular distortion and electronic repulsion between the closely positioned benzene rings.^[13]^

Due to its unique electronic structure and ring distortion, pCp demonstrates heightened basicity/nucleophilicity, enhancing its electrophilic substitution and ability to form p‐complexes.^[14]^ Despite these intriguing properties, synthesizing pCp and its derivatives remains challenging, requiring harsh conditions for cyclization and resulting in an uncontrollable mixture of products during post‐functionalization.[^2^, ^15^] Despite that [2,2’] dibromo paracyclophane isomers are separable and are the main platform for [2,2] cyclophane based molecules. The presence and reactivity of the halogen is crucial for plenty of reactions, among whom pallado‐catalysed couplings.^[16]^ This strategy inspired our synthetic efforts, as presented below.

Another synthesis route,^[17]^ frequently mentioned in various articles, outlines a simpler method for producing pCp. This method revolves around a crucial precursor known as dithia[3.3]paracyclophane (dtpCp). Since the 1970s, this latter is instrumental as a precursor for synthesizing paracyclophane (pCp). This novel synthesis approach involves coupling 1,4‐bis[halomethyl]benzene with 1,4‐bis(mercaptomethyl)benzene, followed by a photodesulfurization step in triethylphosphite. This method enables smoother dtpCp synthesis and in addition, it allows a pre‐functionalization of precursors for creating asymmetric cyclophanes. Sulfur atoms in dtpCp act as hinges, increasing atom mobility and expanding the inter‐ring distance between benzene rings from 3.04 Å for pCp to about 3.24 Å for dtpCp.^[18]^ Despite this inter‐ring distance increase, π‐conjugated system overlap persists in dtpCp derivatives, supporting π‐π transannular interactions seen in fluorene‐dithiaparacyclophane copolymers by Wang et al.,^[19]^ as evidenced through observed emission shifts and improved quantum yield of photoluminescence. While dtpCp synthesis is more accessible than pCp, the π‐π transannular interactions are slightly reduced but never entirely eliminated, suggesting diverse potential applications akin to pCp.

As mentioned earlier, pCp main feature is the two benzene rings forced in close proximity. As well as its chemistry, the π‐π transannular interactions also strongly influence the light matter interaction. Despite this very peculiar architecture only a few studies were published.^[20]^ Anthracenophane was stydied in the 70s by Hayashi *et al*. as a way to promote or hinder its photo‐dimerization.^[21]^ Several studies by Nurmukhametov. *et al*[^22^, ^23^] were focused on deeper understanding of the relationship between the [2,2’]paracyclophane orbital and its photophysics properties. Their study as well as other report^[24]^ on [3,n]cyclophane claimed that the fluorescence is ruled by a strong excimer emission. Cyclophane can also be viewed as forced dimer and notably for stilbene, which cyclophane equivalent was shown to have different isomerization dynamics than the free one.^[25]^

**Figure 1:**
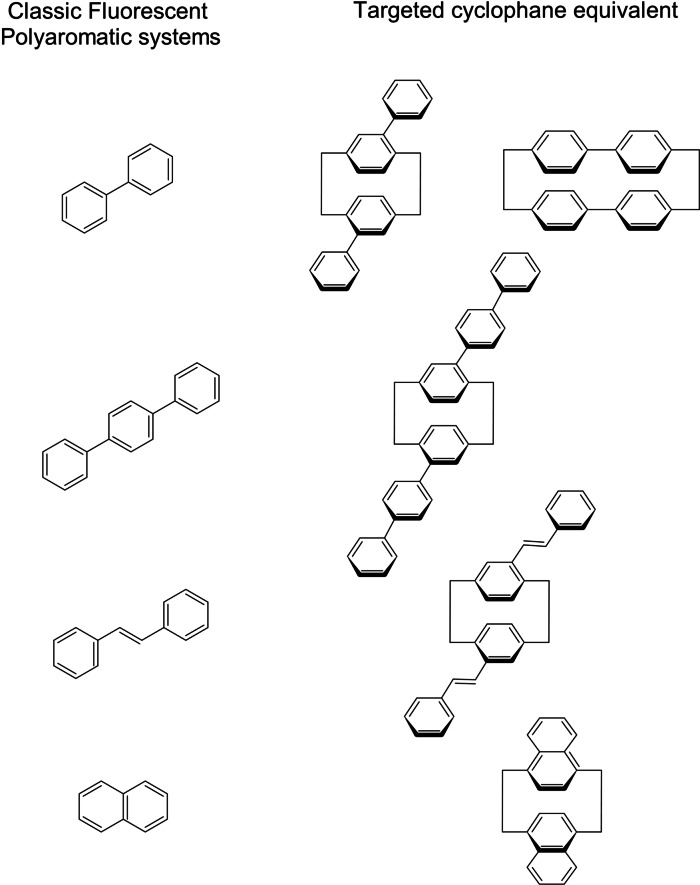
Targets of this study.

All these separate works were made on isolated molecules. We didn't find a comprehensive comparison on different [2,2’]paracyclophane structure that would generalize on their photophysical properties, maybe because of the synthetic challenge such molecules represent. We would like to palliate this fact and to present a comprehensive synthetic and photo physical work on polyaromatic cyclophanes. Thus, in this work, we propose to synthesize a series of molecules based on [2.2] paracyclophane with extended aromatic systems, in order to present a unique reference for later cross‐comparison **(**Figure 1). The focus of our study is their photophysical properties in solution as well as solid state. We expect that pCp based fluorophores can take advantage of the strain geometry to favor specific excited state. We also think that the π‐π transannular interactions will favor exciplex‐like behavior. We also demonstrate the possibility to embed these molecules in plastic films and then get a long‐lasting solid‐state sample.

## Results and Discussion

### Synthesis

**Figure 2:**
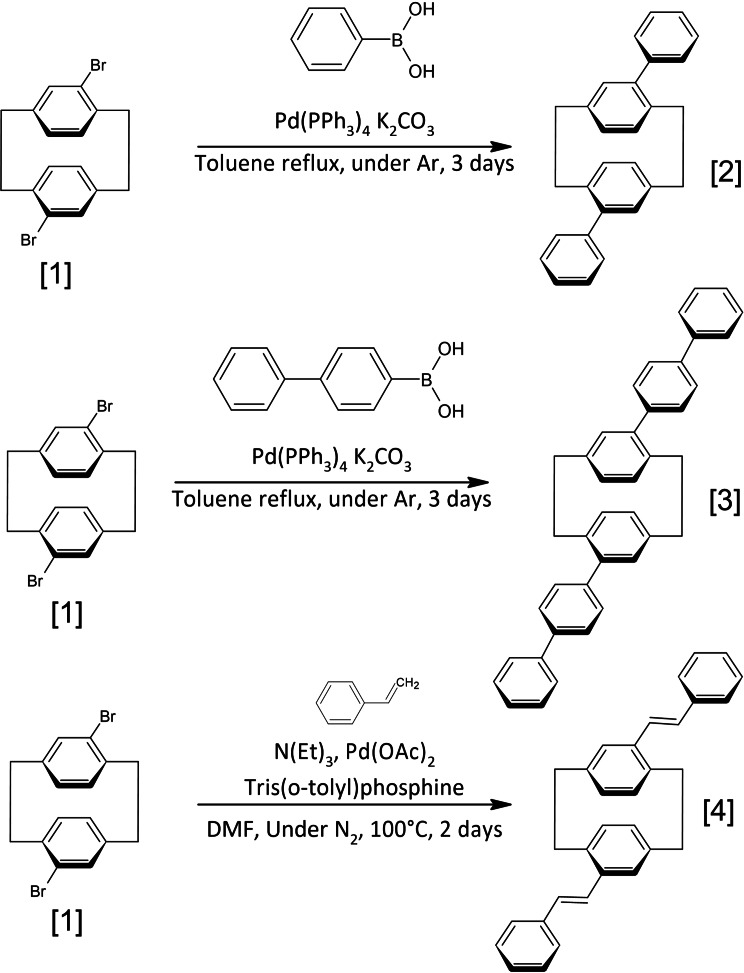
paladocatalysed coupling strategy to obtain polyaromatic paracyclophanes.

The first target compounds are based on pCp, which is commercially available. The dibromination of the latter, described in the literature,^[15]^ is carried out under conditions of high DCM concentration using dibromium and metallic iron as catalyst.^[12]^ This electrophilic substitution reaction can produce several overbromination and degradation products, accentuated by the increased reactivity of strained cyclophane. For this reason, we preferred to work with commercial 4,16‐dibromo[2,2]paracyclophane (Figure 2). The method traditionally used for the synthesis of aryl‐substituted [2.2]paracyclophane derivatives involves the cross‐coupling of monobrominated [2.2]paracyclophane with arylmagnesium bromides catalyzed by nickel phosphine complexes. However, this method often leads to low yields.^[26]^ On the other hand, the advantage of substituting cyclophane with bromine is that coupling reactions can then be easily carried out. Palladium‐catalyzed cross‐couplings can be used for the synthesis of various π‐conjugated molecules and polymers through sp^2^ and sp carboncarbon bond formation.^[27]^

4,16‐diphenyl[2,2]paracyclophane **2**, formed by palladium tetrakis triphenylphosphine‐catalyzed coupling of **1** and phenyl‐boronic acid is carried out in toluene which has been degassed by several cycles of argon vacuum in order to eliminate all traces of dioxygen, the activated catalyst being very sensitive to oxidation. Compound **2**, synthesized by the Suzuki method,^[28]^ is then purified by sohxlet in chloroform, followed by recrystallization in toluene. The 55 % yield obtained can be explained by the steric hindrance inerrant to cyclophane **1** (ESI 2‐a). This may be accentuated by the interconversion of **1** under high thermal activation, resulting in the rotation of the two aromatic rings relative to each other. There is a transition from a pseudo‐para to a pseudo‐ortho relationship between the two bromines in a similar ratio. According to 2D NMR analysis, only one product is present after coupling, and this is indeed a pseudo‐para relationship between the phenyls. Compound **3** is prepared by the same procedure, this time using 4‐biphenyl‐boronic acid achieving a similar yield of 50 %, also using a Soxlet apparatus for purification (ESI 2‐b).

The coupling of styrene with specific bromoparacyclophane derivatives via palladium mediation offers an efficient pathway to create styrylcyclophane compounds. Although the conventional Heck catalyst system[^29^, ^30^, ^31^] used to achieve molecule **4**, comprising a blend of Pd(OAc)_2_ and P(o‐tol)_3_, effectively initiates this reaction, yields typically fall below 50 % when isolated.^[32]^ In fact, steric hindrance around the styryl groups of pseudo‐o‐disubstituted [2.2]paracyclophane during polymerization leads to a poor reactivity toward the Heck protocol. Enhanced yields are achieved using Jeffery's phase‐transfer catalyst system,^[33]^ involving a blend of Pd(OAc)_2_, excess K_2_CO_3_, and NBu_4_Br. Notably, this synthetic technique has been previously utilized by de Meijere and colleagues in producing styryl[2.2]paracyclophane from 4‐bromo‐[2.2] paracyclophane.^[34]^ Additionally, Wittig chemistry has demonstrated utility in the synthesis of compounds of a similar nature^[35]^ (ESI 2‐c).

Cram^[36]^ first reported the synthesis of anti‐2.2naphthalenophane **7** in 1963, which was later refined by Brown and Sondheimer.^[37]^ In 1969, Wasserman and Keehn^[38]^ detailed the synthesis of syn‐2.2naphthalenophane, followed by its thermal conversion to anti‐2.2naphthalenophane upon melting. All these papers present a synthesis protocol involving a coupling reaction between 1,4‐bis(bromomethyl) naphthalene and 1,4‐bis(mercaptomethyl) naphthalene, to give a mixture of syn and anti isomers of 2,15‐dithia[3.3](l,4)naphthalenophane.^[39]^

Our synthetic strategy is a little different, but aligned with the general procedure recently outlined for preparing cyclophanediene compounds in 2 steps:

- The first step is the reaction between 1,4‐dimethylnaphthalene **5**, a brominating agent, and a catalyst in carbon tetrachloride to produce 1,4‐bis(bromomethyl)naphthalene **6** in more than 90 % yield after recrystallization (ESI 2‐d)
- Next, 1,4‐bis(bromomethyl)naphthalene was extracted (Soxhlet) into a refluxing solution of sodium iodide in acetone for 24 hrs. The solvent was evaporated on a rotary evaporator until the overall reaction mixture volume was around 50 ml, and then the mixture was poured into water. Sodium thiosulfate was added until the color changed to white followed by filtering and drying. Recrystallization from dichloromethane furnished pure [2.2](1,4)‐naphthalenophane **7** in 50 % yield as shown in Figure 3
  **Figure 3:**
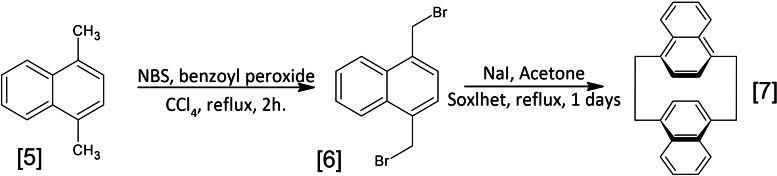
Synthesis of [2.2’](1,4)‐naphthalenophane.
  and ESI 2‐e.

Retrosynthesis of **12** can be envisaged in a variety of ways, with disconnections between aromatic biphenyl units or in aliphatic chains. Moreover, a linear synthesis is also conceivable, ending with macrocycling. However, we opted for a convergent approach, more economical in terms of steps, starting with 4,4′‐dimethylbiphenyl **8**. Compound **9** is obtained by radical dibromination with NBS in CCl4.^[40]^ After purification by silica column chromatography, a fraction of the product can then be used to synthesize **10** by disulfurizing with thiourea in refluxing ethanol. Macrocyclization is then carried out by double nucleophilic substitution under pseudo‐infinite dilution conditions, in order to limit intermolecular reactions once the first substitution has been carried out. A solution of **9** and **15** in DCM is added dropwise to a large volume of methanol basified with KOH. After washing, 2,17‐dithia[3,3](4,4′)‐biphenylophane **11** is obtained by recrystallization in DCM in 73 % yield (ESI 2‐g).

**Figure 4:**
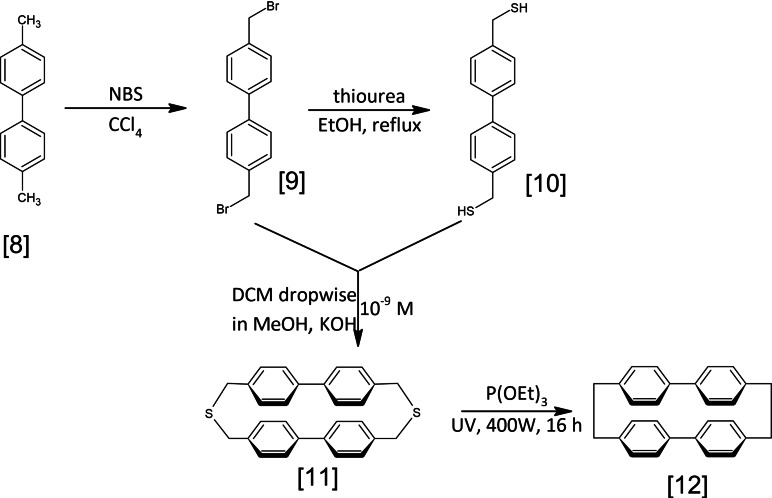
Synthesis of [4,4′]biphenylophane.

The decisive step is the removal of sulfur to reach **12**. The method employed is a photodesulfurization of **10** under irradiation of a triethyl phosphite suspension of with a 400 W high‐pressure lampp.^[11]^ This reaction is nevertheless a limiting step from the point of view of increasing the quantities of material involved. Irradiation area and solution homogeneity are strongly correlated with reaction rate. An alternative would be to use a benzyne to form the zwitterionic intermediate capable of rearranging itself according to Stevens [1,2] rearrangement.[^41^, ^42^] The product can then be reduced to give **12 (**Figure 4) (ESI 2‐f).

With these five different poly aromatic cyclophane systems, we then turn our interest towards their photophysics. We are especially curious of the comparison between the independent well‐known polyraromatic fluorophore and their cyclophane equivalent.

### Photophysics

Solutions for fluorescence measurements were prepared to specific concentrations such that the maximum absorbance was ∼0.1. The solutions were degassed and introduced into a quartz cuvette equipped with a Teflon valve in order to minimize the influence of oxygen on the TCSPC measurements. Fluorescence was measured at right angles using a 1 cm cuvette. We decided to measure absorption and emission either in toluene or in dichloromethane, in order to determine if the pCp‐based fluorophores could present any solvato‐chromism and/or if their behaviour is comparable to their corresponding homologues.

**Figure 5:**
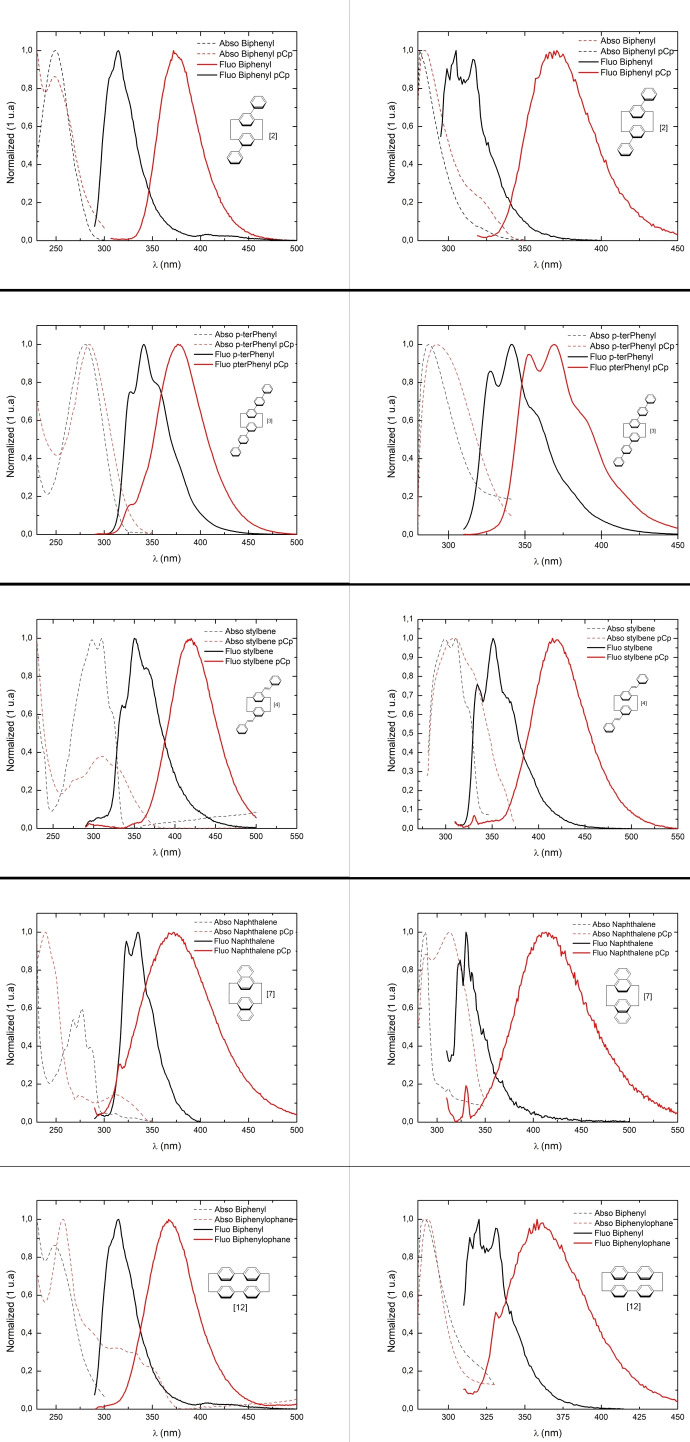
Absorption (in dash line) and emission (in plain line) of our five cyclophane (red) and their non‐cyclophane equivalent (black). Left column in dichloromethane, right column in toluene. (secondary pick around 330 nm are parasite Raman pick).

Figure 5 shows us several constant feature. For biphenyl and terphenyl based cyclophanes **2** & **3**, the same absorption profile is observed compared to their non cyclophane equivalents, this hint us as an absorption process that is independent of the cyclophane. Stylbene and biphenylophane derivatives **4** & **12** present some discrepancy in the intensity but same fine structure when observed in in DCM, but show similar absorption profile in toluene, hinting here also to an absorption process with minimal influence of the cyclophane unit. Anthracene derivative **7** is an exception to this trend with a strongly red shifted absorption, both in toluene and DCM. This could indicate an absorption process through the Cyclophane unit.The fluorescence, in all case, is red shifted. The similar absorption and the red shifted fluorescent make the inclusion of a cyclophane a consistent strategy for designing large Stockes shift emitters.

**Figure 6:**
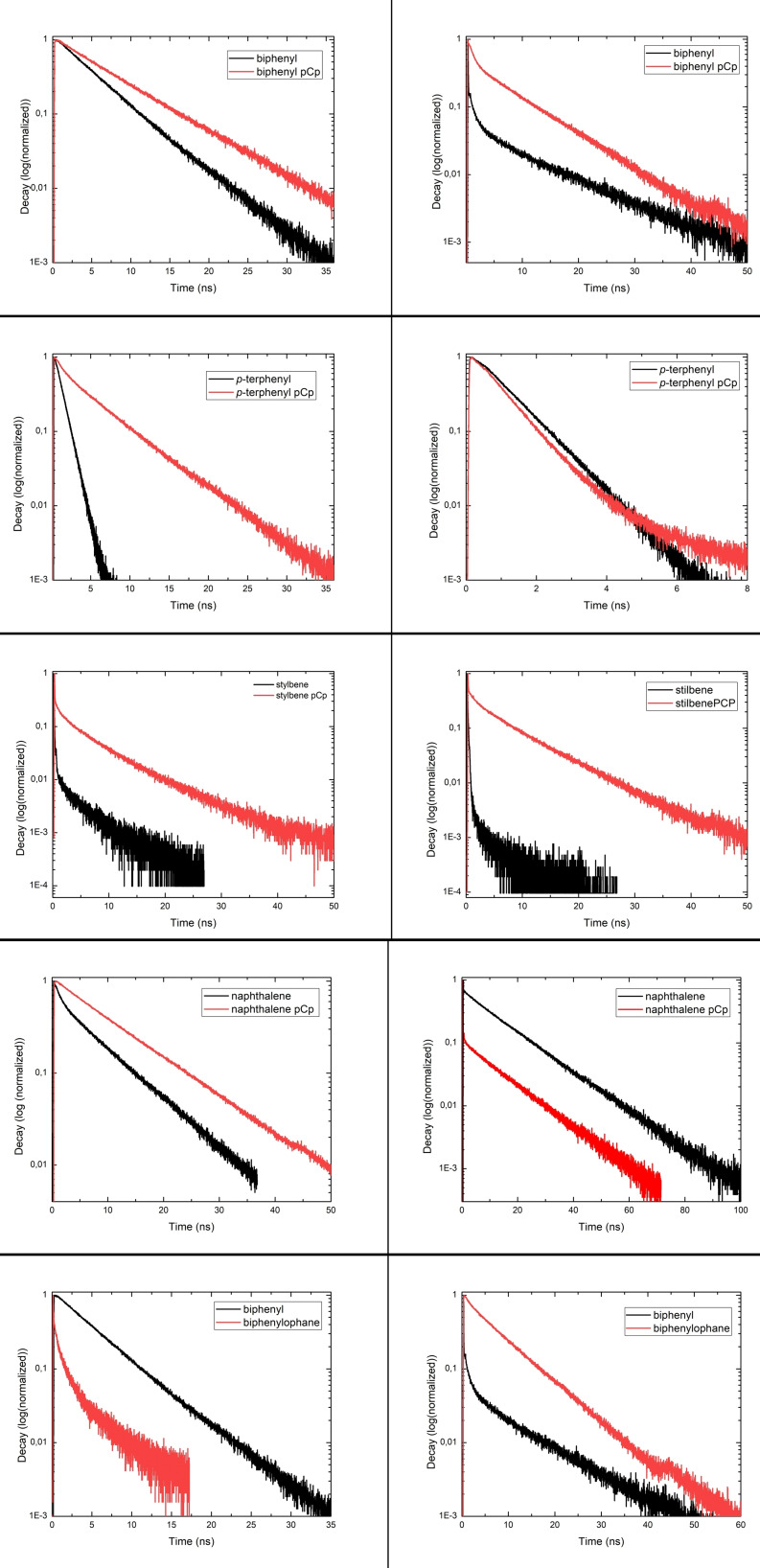
TCSPC decay time of our five cyclophane (red) and their non‐cyclophane equivalent (black). Left column in dichloromethane, right column in toluene.

The fluorescence's shape is nevertheless modified. Most of the homologue molecules present a fine structure coming from discrete fluorescence process. All cyclophane present a larger full width at half maximum (FWHM) of the fluorescence and a loss of this fine structure, which is attributed to excimer based emission.[^22^, ^23^] Only p‐terphenyl equivalent present some retention of the structure. This could be due to a more extended aromatic system, which delocalize the excited state and diminish the influence of the cyclophane subunit. This set of experiment also show that the incorporation of cyclophane does not influence the solvatochromism properties. This indicate that, at first glance, cyclophane incorporation does not change the solvation sphere influence on the absorption/emission process for molecules [2], [3], [4] and [12]. The naphthalene derivative [7] is an exception here with an emission at 370 nm in DCM and 413 nm in toluene. As a complement, and in order to understand the photophysics of our pCp‐based fluorophores, we compared their decay time in different solvents to their corresponding homologues. Hence, we performed time‐correlated single photon counting TSCPC experiment, presented in Figure 6.

The results show discrepancy between solvents and no consistent behaviour, hinting as a more complex role of the solvant spheres and the cyclophane moiety than expected. The only trend that jumps out is that pCp fluorescence are generaly slower than their equivalent. We postulate that the geometry of the pCp hinder planar molecular configuration that could lead to fast fluorescence, hence quenching the fast decay, as opposed to molecule [12] were the planar configuration is forced. This could be also linked to a more stable excited state and in some case a forced exciplex structure. Two exceptions at this trend are naphthalene pCp in toluene, and the biphenylophane in DCM. The first dependency shows us the pCp fluorescence process can rely on external parameter as was put forward for the solvent dependency od naphthalene pCp fluorescence. We postulate that toluene can act as supplementary pi‐pi donor, forcing different geometry of the pCp molecules, hence the spread of observed decay time.

The result from the p‐terphenyl pCp highlights the solvent role in this experiment as there is a large discrepancy of behevior. The fact that decay time in toluene is almost identical could be linked to our previous observation. It concurs with our fluorescence observation of a more delocalized excited state farther away from the cyclophane sub‐unit, hence minimizing the cyclophane moiety influence on the pCp‐based fluorophore properties. However, this is not what we observe in DCM as *p‐*terphenyl emission remains fast, whereas the *p‐*terphenyl pCp slows down greatly.

We complemented those observations with measuring the quantum yields of our five molecules (Table 1). Here also with have no definitive trends. Stilbene and biphenyl seem to improve greatly when converted in cyclophane whereas *p‐*terphenyl naphthalene loses emission probability.

**Table 1:** Quantum yields of our molecules in DCM.

Molecules	QY
Biphenyl	5 %
Biphenyl pCp [2]	6 %
Biphenylophane [12]	11 %
*p‐*terphenyl	34 %
*p‐*terphenyl pCp [3]	9 %
Stilbene	4 %
Stilbene pCp [4]	22 %
Naphtalene	18 %
Naphtalene pCp [7]	11 %

The large increase of quantum yields for the biphenylophane [12] is particularly interesting; we postulate it is due to the acceleration of the emission dynamics observed by TSCPC. One explanation could be that biphenylophane blocked completely the rotation in the biphenyl sub‐unit. This prevents consequently warped geometry that favour non‐radiative despeciation.

As for Stilbene pCp, *cis‐trans* photo‐isomerisation is a main factor of its low quantum yields. The addition of cyclophane moiety hinder the isomerization and increase a radiative outcome for an excited state.

One main concern for the utilisation of pCp based molecules as dyes or fluorophores resides in their poor solubility. Despite that it is possible to incorporate our five molecules in a polystyrene (PS) matrix. We manufactured films containing an equivalent concentration as our liquid state photophysics experiments. PS plastic can be seen as solid‐state solution, with remarkable lifetime, transportability without spilling risk and easily sharable.

**Figure 7:**
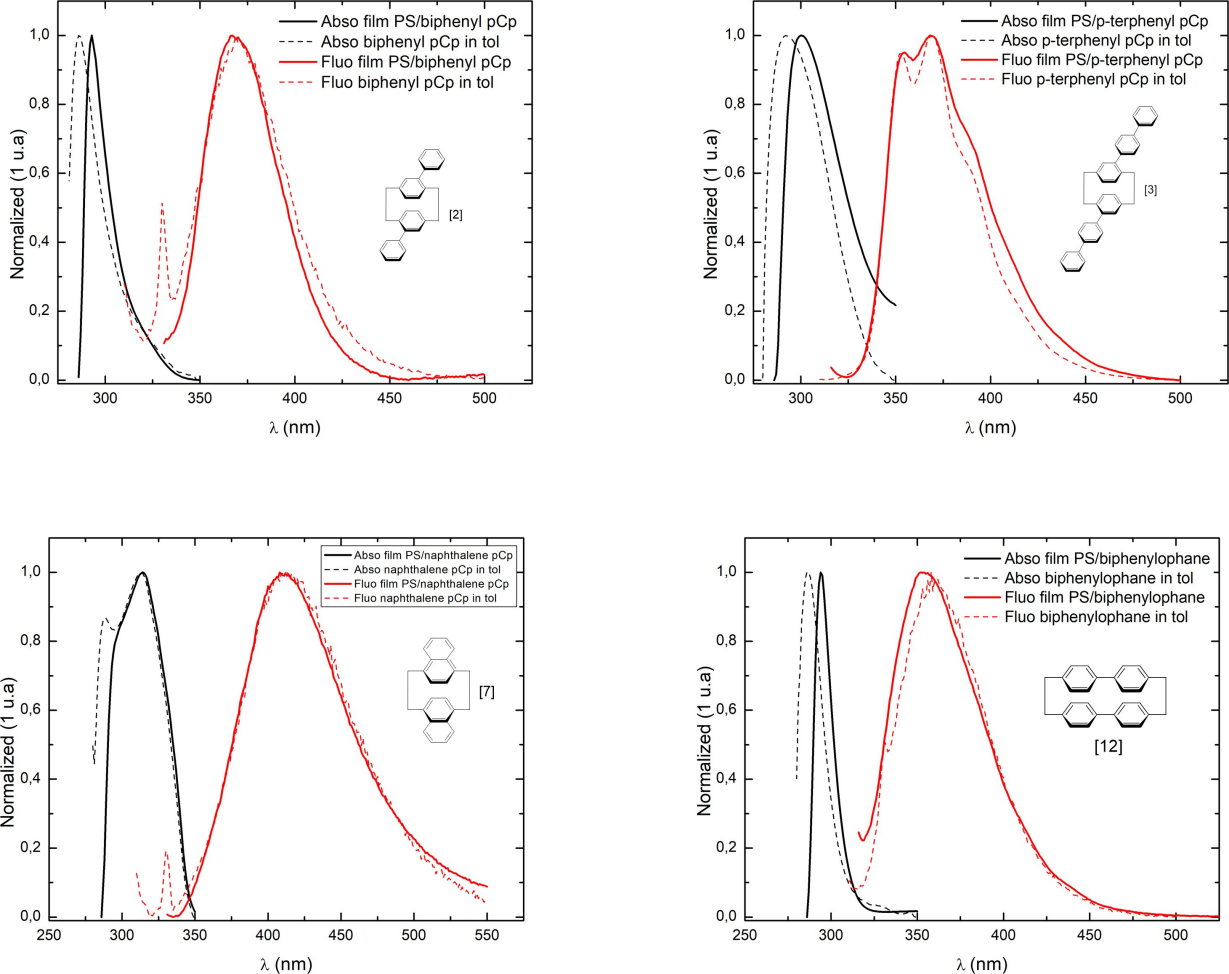
Absorption and fluorescence spectra of molecules [2], [3], [7] and [12].

The result are presented in Figure 7. We observe little difference for the photophysics properties at the exception of the disappearance of the Raman parasite peak. PS is thus a valid and long lasting medium for our studies. This experiment shows us that polystyrene film can be consider as a solid solution medium for pCp optical properties. This is to link with precedent studies that have also demonstrated that PMMA films containing molecules possessing a luminogenic silole and chiral sugar groups,^[43]^ along with bischromophoric derivatives of perylene bisimide^[44]^ or [2.2]Paracyclophane‐based double helices,^[45]^ maintain significant optical properties compare to solution, including circularly polarized luminescence (CPL) with high dissymmetry factors and elevated quantum yields. These findings emphasize the feasibility of incorporating paracyclophane‐based molecules into PMMA films, similar to polystyrene, thereby advancing the development of novel chiral organic materials with outstanding performance for various applications in optoelectronics and information processing.

## Conclusions

We present here individual synthetic route for five different polyaromatic cyclophane based molecules. Pallado‐catalized coupling is a valid strategy when starting with the commercial [2,2’] dibromo paracyclophane, as a primary platform for extended aromatic system. We also show a way to synthesize [2.2’](1,4)‐naphthalenophane and use the recently published dithyacyclophane approach to obtain biphenylophane. All cyclophanes molecules were synthesize with moderate yield and showed identic NMR to previously reports. Each molecules was then characterized in absorption, emission, quantum yields and decay time in different solvents as well as embed in solid plastic films, in order to show the influence of the cyclophane on the photophysics properties. We notably show a general increase in the Stockes shift as well as a general increase of the emission decay time, which is coherent with existing litteratures. For all these experiments, we show a major role of the solvent on the photo physical process. We underline an increase of the fluorescence quantum yields for Biphenylophane and styrene pCp. Despite their overall poor solubility, it is possible to manufacture polystyrene film loaded with our cyclophane‐based molecules. We expect that this approach will lead to new opportunity in chemical design of fluorescent molecules. one of our main perspective is indeed to explore more in depth their photophysics with transient spectroscopy in order to explore possible orbital communication in the [2,2’] paracyclophane sub unit.

## Conflict of Interests

The authors declare no conflict of interest.

1

## Supporting information

As a service to our authors and readers, this journal provides supporting information supplied by the authors. Such materials are peer reviewed and may be re‐organized for online delivery, but are not copy‐edited or typeset. Technical support issues arising from supporting information (other than missing files) should be addressed to the authors.

Supporting Information

## Data Availability

The data that support the findings of this study are available from the corresponding author upon reasonable request.
