# Supplementary material for: Polyaromatic Cyclophanes Design and their Related Optical Properties
Source: ChemistryOpen. 2024 Dec 4;14(4):e202400207. doi: 10.1002/open.202400207 (PMC11973505; doi:10.1002/open.202400207)

ChemistryOpen

Supporting Information

Polyaromatic Cyclophanes Design and their Related Optical Properties

Oumou Diallo, Jean-Frédéric Audibert, Isabelle Leray, David Kreher,* and Guillaume H. V. Bertrand*

SUPPORTING INFORMATIONS

Polyaromatic cyclophanes design and their related optical properties.

Oumou DIALLO[a][b], Jean-Frédéric AUDIBERT[c], Isabelle LERAY[c], David KREHER[a]* Guillaume H.V. BERTRAND[b]*,

a] Institut Lavoisier de Versailles (ILV), CNRS, Université Paris-Saclay, 45 avenue des Etats-Unis, F-78035 Versailles, France, oumou.diallo@uvsq.fr; david.kreher@uvsq.fr.

[b] [Université Paris-Saclay, CEA, List, F-91120 Palaiseau, France, oumou.diallo@cea.fr; guillaume.bertand@cea.fr.

[c] Université Paris-Saclay, ENS Paris-Saclay, CNRS, Photophysique et Photochimie Supramoléculaires et Macromoléculaires, 91190, Gif-sur-Yvette, France

1) General and Materials Information

a. Chemistry

All anhydrous solvents for syntheses and starting chemicals were purchased from commercial suppliers (Aldrich or Acros) and used without further purification. [2,2'] dibromo paracyclophane was purchase from Ambeed ad Fisher Scientific. All glassware was oven-dried and cooled under an inert atmosphere of argon. All air sensitive reactions were carried out under argon atmosphere using standard Schlenk techniques. Flash column chromatography was performed on silica gel (200-300 mesh).
All presented synthesis were the most conclusive one among all the condition tested.
1H NMR (200 or 300 MHz) and 13C (50 or 75 MHz) spectra were recorded with Bruker DPX 200 MHz, or Bruker Avance 300 MHz spectrometers using tetramethylsilane as an internal standard. Chemical shifts (δ) are given in parts per million and coupling constants are given as absolute values expressed in Hertz. Data are reported as follows: chemical shift, multiplicity (s = singlet, d = doublet, m = multiplet), coupling constant (Hz), and integration. High-resolution mass spectrum was acquired using MALDI-TOF-MS techniques.

b. Photophysics

Solutions for fluorescence measurements were prepared using spectroscopy grade solvent purchased from Carlo Erba to specific concentrations such that the maximum absorbance was ~ 0.1. The solutions were introduced into a quartz cuvette equipped with a Teflon needle valve to minimize contact with oxygen. Fluorescence was measured at right angles using a 1 cm cuvette. The absorption spectra were recorded by using double-beam spectrophotometers Cary 4000 or Cary 5000 from Agilent Technologies. The emission spectra were measured using Horiba Jobin-Yvon spectrofluorometer Fluorolog FL3-221. The fluorescence quantum yield were determined by relative quantum yield measurement with naphthalene in cyclohexane as a standard sample (literature $\Phi_F(0) = 0.23$).

Fluorescence decay curves were obtained by the time-correlated single-photon counting (TCSPC) method. The setup is composed of a titanium sapphire Ti:Sa LASER (Spectra Physics, Maï Taï HP) emitting pulses of 100 fs duration at 780 nm, 80 MHz frequency. The laser pulses then pass through a pulse picker to reduce the repetition rate at 4 MHz. The signal is tripled between 270 nm to 300 nm by focalizing the laser in a non-linear SHG-THG crystals (GWU Lasertechnik, UHG-23-PSK). Then the beam passes through the sample solution after adjusting the excitation power with an intensity attenuator filter wheel. Fluorescence photons are detected at 90° through a monochromator (HORIBA,MicroHR) and a polarizer at magic angle by means of a micro channel plate photomultiplier (Hamamatsu, MCP-PMT R3809U-50), connected to a TCSPC module (Becker & Hickl, SPC-130-EMN). The instrumental response function is recorded before each decay measurement with a fwhm (full width at half maximum) of ~25 ps. Time-correlated fluorescence decay data are finally processed and analyzed with the help of a software which implements the non-linear square method (Globals, Laboratory for Fluorescence Dynamics at the University of California, Irvine).

c. Polymer films

Doctor-blading was used to produce thin films composed of PS, biphenyl pCp, p-terphenyl pCp, naphthalene pCP and biphenylophane. The formulation consisted of a ratio of 30 mg pCp to 7 g PS with 11 ml toluene. The entire mixture was mixed in a planetary mixer for three cycles, each lasting 30 minutes.
During this process, a precise blade was positioned at a fixed distance above the substrate surface (800 µm). The formulated mixture was then carefully placed in front of the blade. The blade, following a linear path over the substrate, deposited a wet film, which was then dried to form the desired thin film. The final film thickness was achieved at 200 micrometers, with a minimum deviation of ± 5 micrometers. This meticulous process produced consistent, controlled thin films for the substances concerned.

2) Cyclophane Synthesis

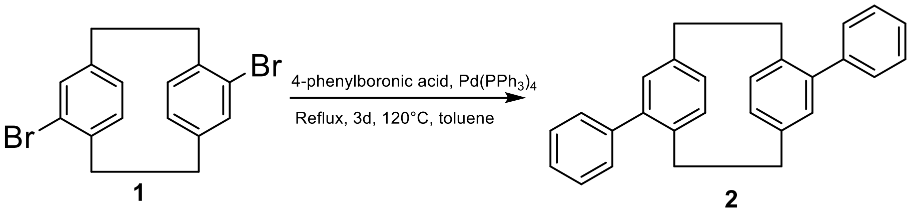

a. **1,4-diphenyl-[2,2]paracyclophane (2)**

To a solution of 4,16-dibromocyclophane 1 (2.196 g, 6 mmol) in 150 ml toluene are added phenylboronic acid (2.925 g, 24 mmol), $Pd(PPh_3)_4$ (0.69 g, 0.6 mmol) and K_2CO_3 (3.32 g, 24 mmol). After 3 days of reaction at 120°C with stirring, the reaction is cooled to room temperature. The solid is then filtered and purified in a soxhlet assembly for 24 hours in chloroform. Evaporate the mixture in vacuo before recrystallizing the solid in toluene. In parallel, recover the filtrate from the first filtration, evaporate the solvent and recrystallize the resulting solid in toluene. TLC in pentane 99:1 AcOEt (Rf=0.33). **2** is obtained as a white crystallized solid (yield: 1070 mg, 55%). ^{1}H NMR (300 MHz, $CDCl_3$): δ 7.53-7.50 (m, 10H), 6.68-6.66 (m, 6H), 3.47-3.40 (m, 2H), 3.07-2.78 (m, 6H). ^{13}C NMR (CD_2Cl_2): 142.4, 141.7, 140.4, 137.3, 135.2, 132.5, 130.2, 129.6, 128.9, 127.2, 34.9, 34.1. HRMS ($C_{28}H_{24}$) calc. 361.1954, found 361.1956. Mp = 287.5 °C

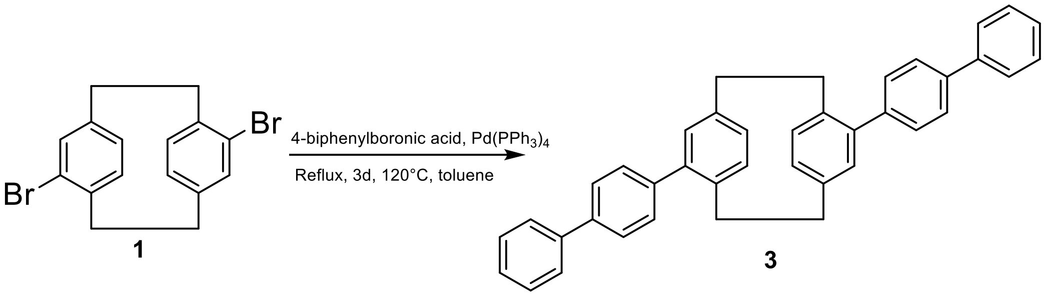

b. **1,4-di([1,1-biphenyl]-4-yl)-[2,2]paracyclophane (3)**

To a solution of 4,16-dibromocyclophane 1 (2.2 g, 6 mmol) in 150 ml toluene are added biphenylboronic acid (4.76 g, 24 mmol), $Pd(PPh_3)_4$ (0.69 g, 0.6 mmol) and K_2CO_3 (3.32 g, 24 mmol). After 3 days of reaction at 120°C with stirring, the reaction is cooled to room temperature. The solid is then filtered and purified in a soxhlet assembly for 24 hours in chloroform. Evaporate the mixture in vacuo before recrystallizing the solid in toluene. In parallel, recover the filtrate from the first filtration, evaporate the solvent and recrystallize the resulting solid in toluene. TLC in pentane 99 :1 AcOEt (Rf=0.33). **3** is obtained as a white crystallized solid. (Yield: 795 mg, 50%). ^{1}H NMR (300 MHz, $CDCl_3$): δ 7.73-7.48 (m, 20H), 6.71 (m, 4H), 3.47-3.40 (m, 2H), 3.55-2.90 (m, 8H). Mp = 137.4°C

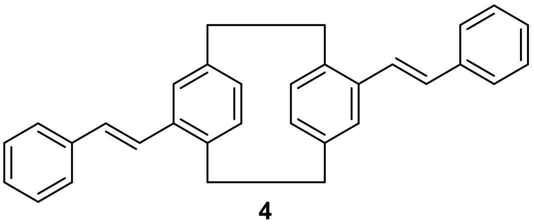

4

c. **4,16-Distyryl[2.2]paracyclophane (4)**

A 100-mL round-bottom flask was charged with a Teflon-coated stir bar, 4,16-dibromoparacyclophane (323 mg, 0.88 mmol), styrene (0.3 mL, 2.6 mmol), triethylamine (2 mL), and DMF (25 mL). The mixture was bubbled with argon for 15 minutes, where Pd(OAc)2 (4 mg, 0.018 mmol) and tris(o-tolyl)phosphine (21 mg, 0.07 mmol) were added to the flask and was bubbled with argon for 15min. The flask was then fitted with a septum (secured with wire) and heated to 100 °C for 2 days while stirring. The cooled reaction mixture was diluted with water to afford a tan precipitate, which was filtered off and rinsed with water. The solid was dissolved in chloroform, filtered into a clean flask, and dried ($MgSO_4$). After filtering off the $MgSO_4$, the chloroform solution was reduced in volume and layered with hexanes to afford 171 mg (47%) of colorless crystals upon standing overnight). TLC in pentane 99 :1 AcOEt (R_f=0.27). ^{1}H NMR ($CDCl_3$): 7.57 (d, J = 7.2 Hz, 2 H, *o*-phenyl); 7.40 (t, J = 7.4 Hz, 2H, *m*-phenyl); 7.28 (t, J = 7.4 Hz, 1 H, *p*-phenyl); 7.21, 6.87 (AB spin system, J = 16.1 Hz, 1 H each, vinyl); 6.68-6.61 (m, 2 H); 6.40 (d,J = 7.7 Hz, 1 H); 3.59 (m, 1 H); 3.11 (m, 1 H); 3.03-2.89 (m, 2 H). ^{13}C NMR ($CDCl_3$): 139.5, 138.2, 137.9, 137.4 (quat.), 133.6, 130.2, 129.5, 129.3, 128.7, 127.5, 127.0, 126.5, 34.5, 33.3. HRMS ($C_{32}H_{28}$) calc. 413.2284, found 413.2269

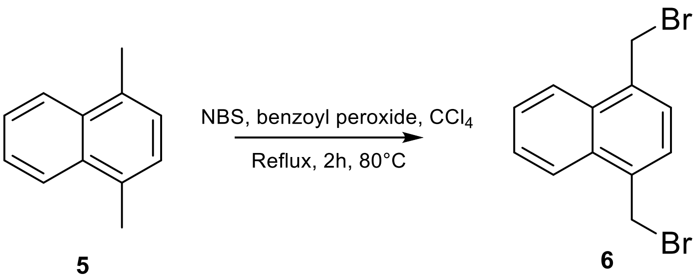

d. **1,4-bis(bromomethyl)naphthalene (6)**

1,4-dimethylnaphthalene 5 (5 g, 30 mmol), NBS (12.1 g, 68 mmol) and benzoyl peroxide (0.16 g) are mixed in CCl_4 (55 ml) at 80°C under reflux for 2h. After allowing the mixture to cool, the solid obtained is filtered and recrystallized in absolute ethanol. TLC in pentane 9:1 DCM (Rf=0.51). A beige solid **6** is obtained (yield: 9.78 g, 97%). ^{1}H NMR (300 MHz, CDCl3): δ 8.22 (m, 2H), 7.67 (m, 2H), 7.49 (s, 2H), 4.94 (s, 4H). ^{13}C NMR (CD_2Cl_2): 135.5, 131.9, 127.7, 127.2, 124.9, 31.8. Mp = 188°C.

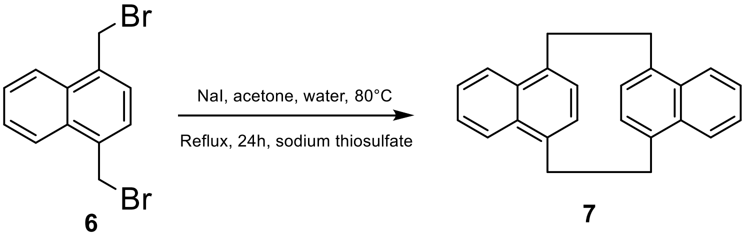

e. **1,4(1,4)-dinaphthalenacyclohexaphane (7)**

Using a soxhlet set-up, mix 2 equivalents of NaI in 150 ml of acetone and add 1 equivalent of 6 to the cartridge. Heat the reaction to 80°C to initiate reflux for 24 hours. Allow the crude to cool to room temperature and evaporate the mixture in vacuo until the approximate volume is 50ml. Then add 150ml of water and, while stirring, gradually add sodium thiosulfate until the mixture turns white. Filter the solid and allow to dry. Recrystallize in dichloromethane or absolute ethanol. TLC in pentane 9 :1 DCM (Rf=0.51). **7** is obtained as a white crystallized solid (yield: 550 mg, 37%). ^{1}H NMR (300 MHz, $CDCl_3$): δ 2.99-3.08 (m, 4H), 3.67-3.83 (m, 4H), 5.76 (s, 4H), 7.39-7.44 (m, 4H), 7.71-7.76 (m, 4H). ^{13}C NMR ($CDCl_3$): 133.4, 132.8, 125.9, 123.6, 123.1, 30.5. HRMS ($C_{24}H_{20}$) calc. 309.1635, found 309.1643. Mp= 299-301°C

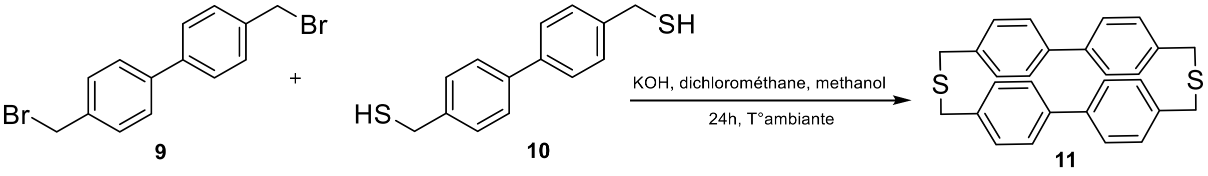

f. **4,9-dithia-1,2,6,7(1,4)-tetrabenzenacyclodecaphane (11)**

Into a 1L flask, place 600 ml of methanol, a pellet of KOH (0.132 g, 2.3 mmol) and a bar magnet. Prepare two solutions: the first containing the two reagents 4,4'-bis(bromomethyl)-1,1'-biphenyl 9 (0.8g, 2.3 mmol) and [1,1']-biphenyl]-4,4'-diyldimethanethiol 10 (0.58 g, 2.35 mmol) in 50 ml DCM and the second containing two KOH pellets (0.264g, 4.7 mmol) in 50 ml methanol. Place these two solutions in 50 ml syringes and, using a syringe pump, introduce them into the flask at a rate of 3ml/h. Work at room temperature. When the reaction is complete, acidify the mixture with 37% HCl or H_2SO_4 from pH 7-8 to pH 1. Then wash the solution with 3x100 ml water and extract in DCM. Dry with $MgSO_4$ and evaporate the solvent in vacuo. Recrystallize the solid and filtrate in DCM. (Yield: 0.735 g, 73%). TLC in pentane 7:3 DCM (R_f=0,5). **11** is obtained as a white solid. ^{1}H NMR (300 MHz, CDCl3): δ 6.97-6.96 (m, 16H), 3.87 (s, 8H). ^{13}C NMR ($CDCl_3$): 139.2, 137.7, 130.5, 126.7, 34.8.

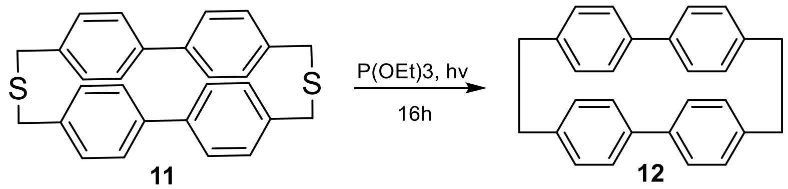

11 **12**

g. **1,2,5,6(1,4)-tetrabenzenacyclooctaphane (17)**

1 equivalent of 11 (400 mg, 0.94 mmol) in 10 ml of triethyl phosphite is introduced into a schlenk tube fitted with a bar magnet. The tube is sealed before being irradiated for 16h under a 400 W UV lamp. The mixture was quenched with 100 ml water and stirred overnight. The reaction was then extracted with dichloromethane, dried with $MgSO_4$ and evaporated in vacuo. The compound is then purified using a chromatographic column with pentane/dichloromethane (7:3) as eluent (R_f=0.9). **12** is obtained as a white solid (yield: 0.1 g, 29%). %). ^{1}H NMR (300 MHz, $CDCl_3$): δ 6.72 (dd,16H), 2.98 (s, 8H). ^{13}C NMR (CD_2Cl_2): 139.0, 137.5, 129.4, 126.6, 38.8. HRMS ($C_{28}H_{24}$) calc. 361.1960, found 361.1956

3) ^{1}HNMR

1,4-diphenyl-[2,2]paracyclophane

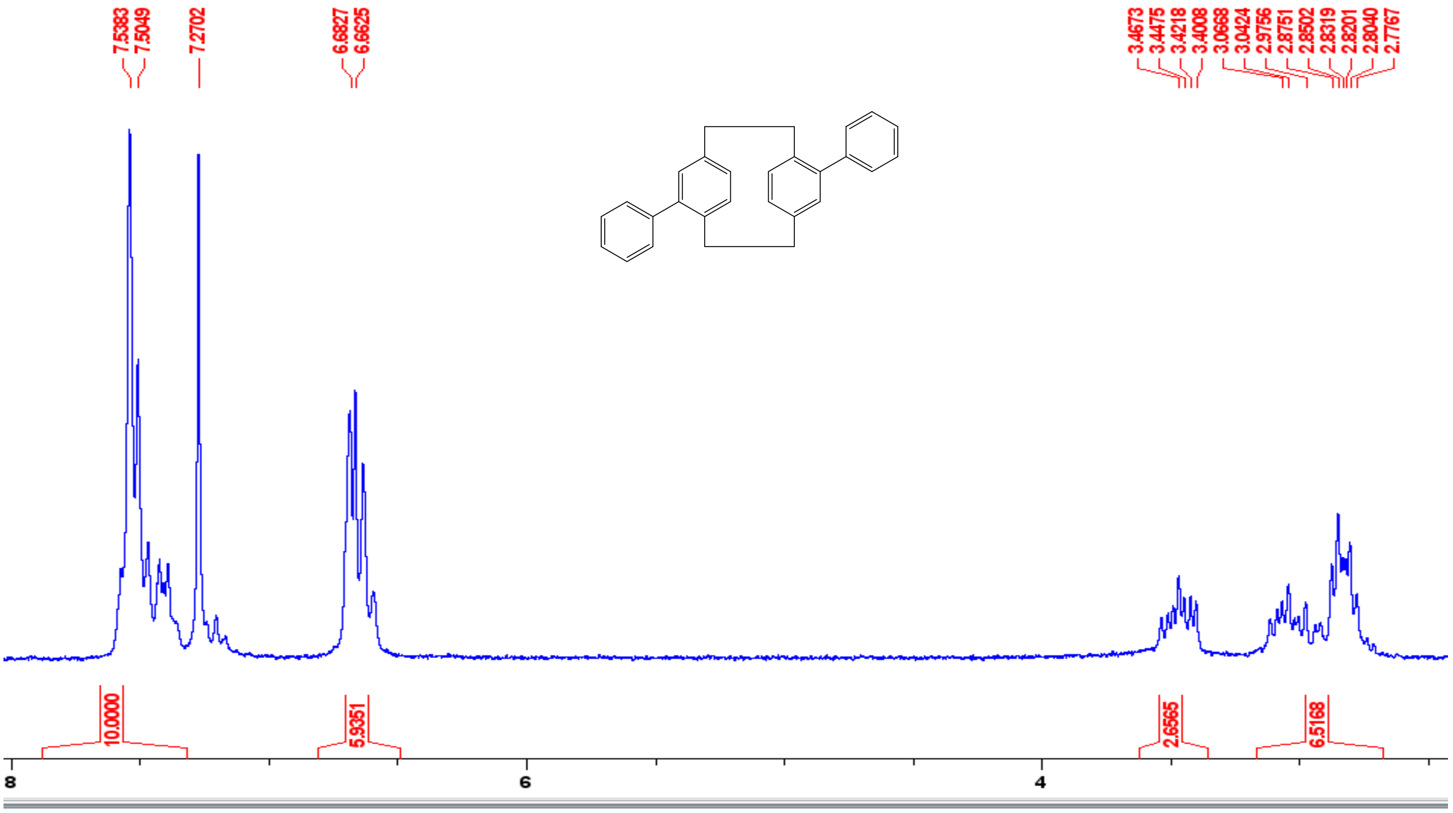

1,4-di([1,1-biphenyl]-4-yl)-[2,2]paracyclophane

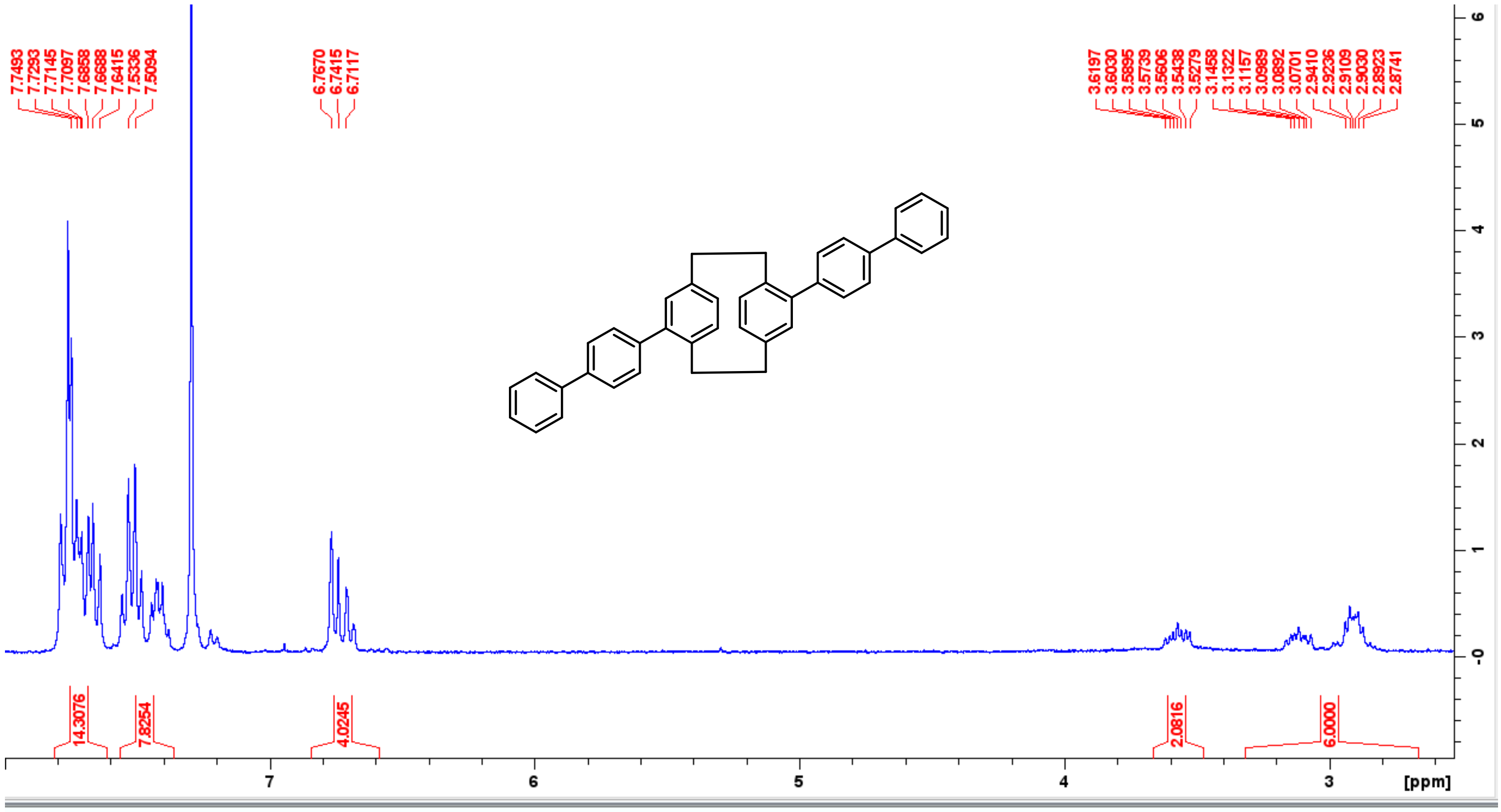

4,16-distyryl[2, 2]paracyclophane

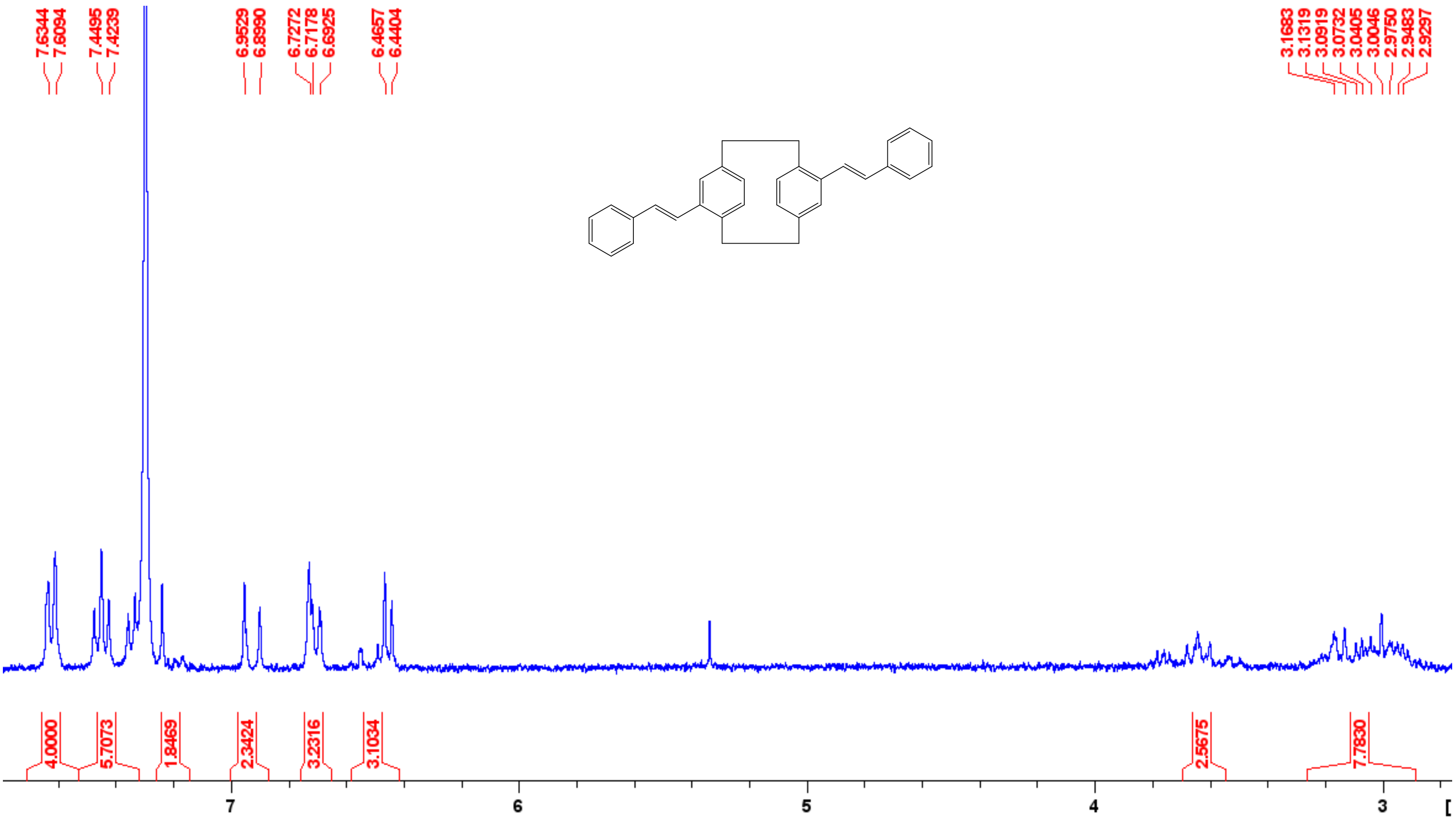

1,4-bis(bromomethyl)naphthalene

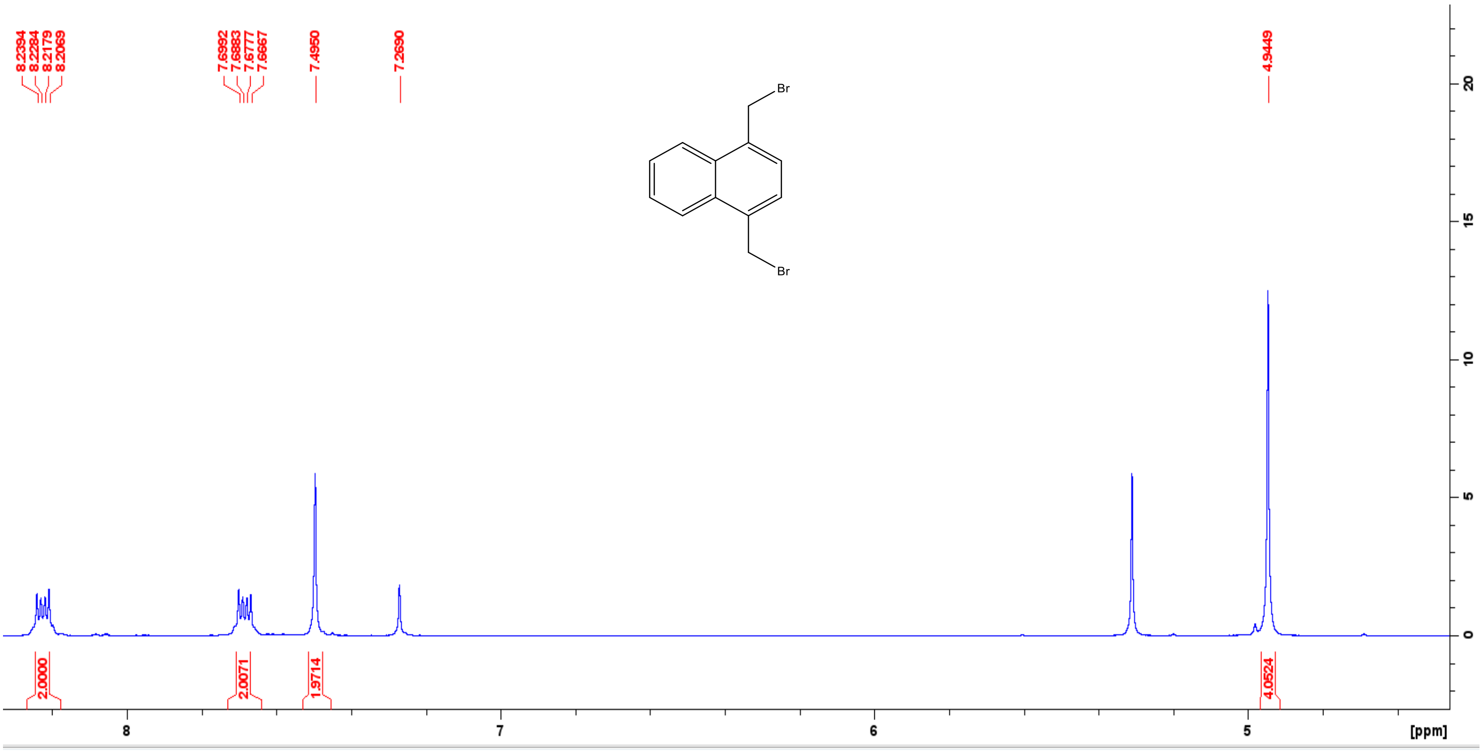

1,4(1,4)-dinaphthalenacyclohexaphane

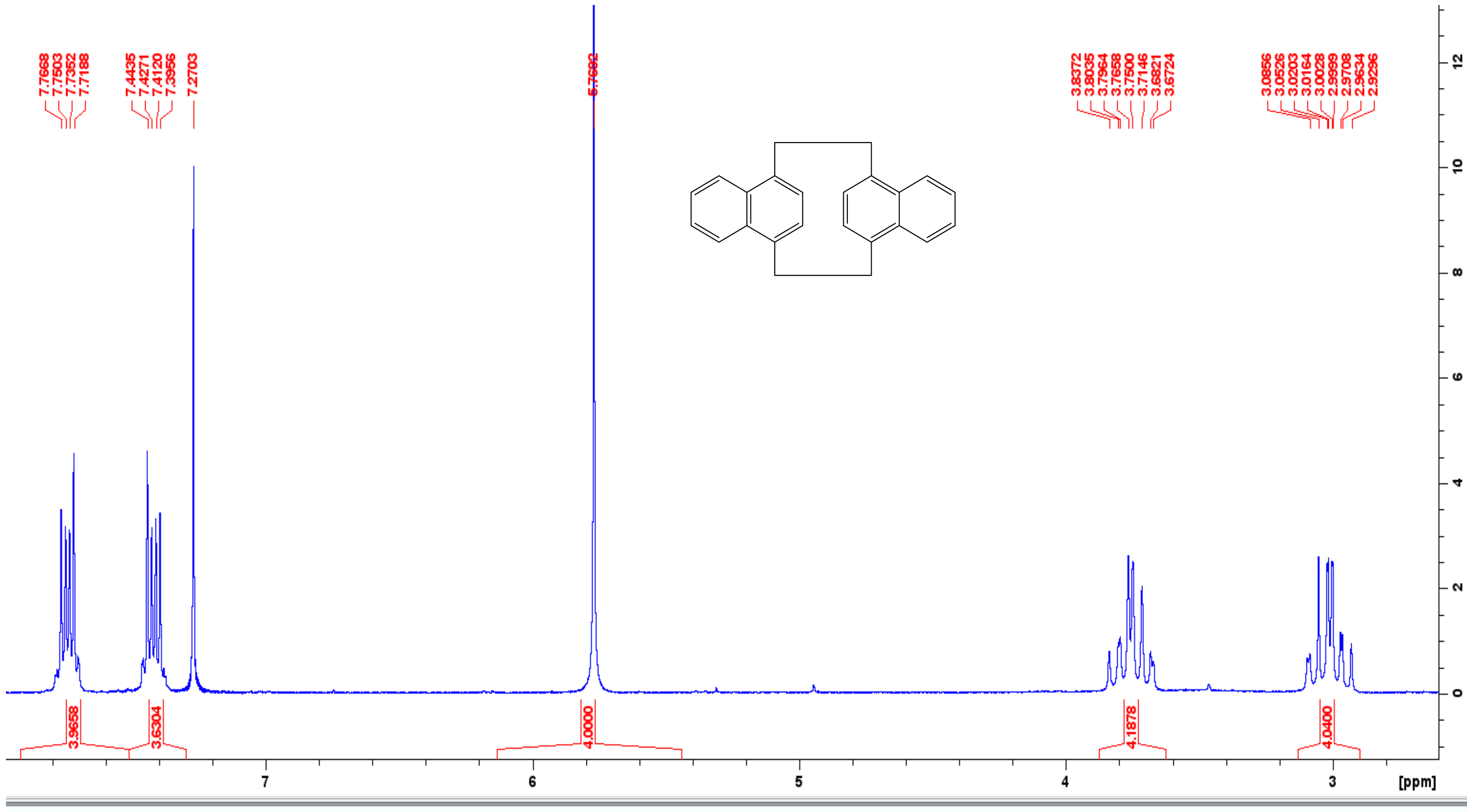

4,9-dithia-1,2,6,7(1,4)-tetrabenzenacyclodecaphane

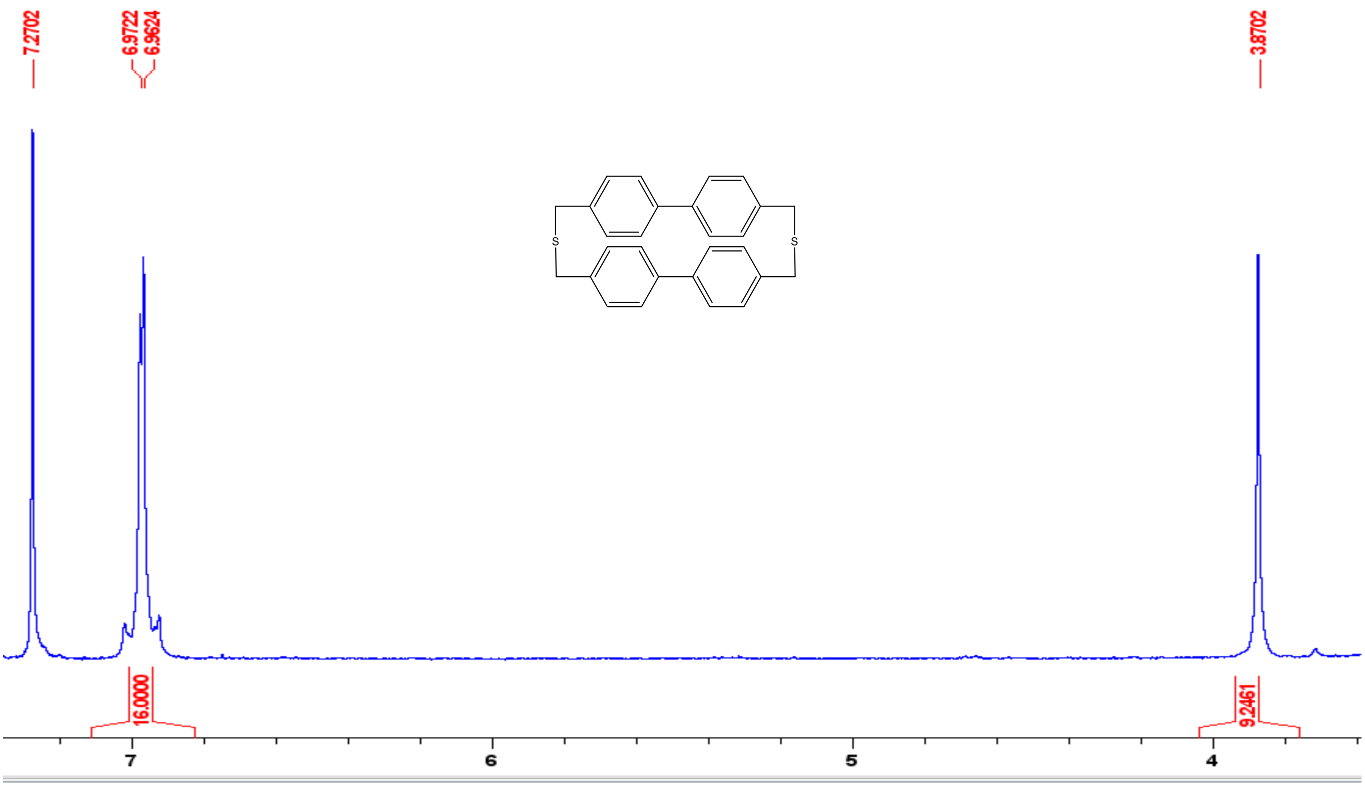

1,2,5,6(1,4)-tetrabenzena cyclooctaphane

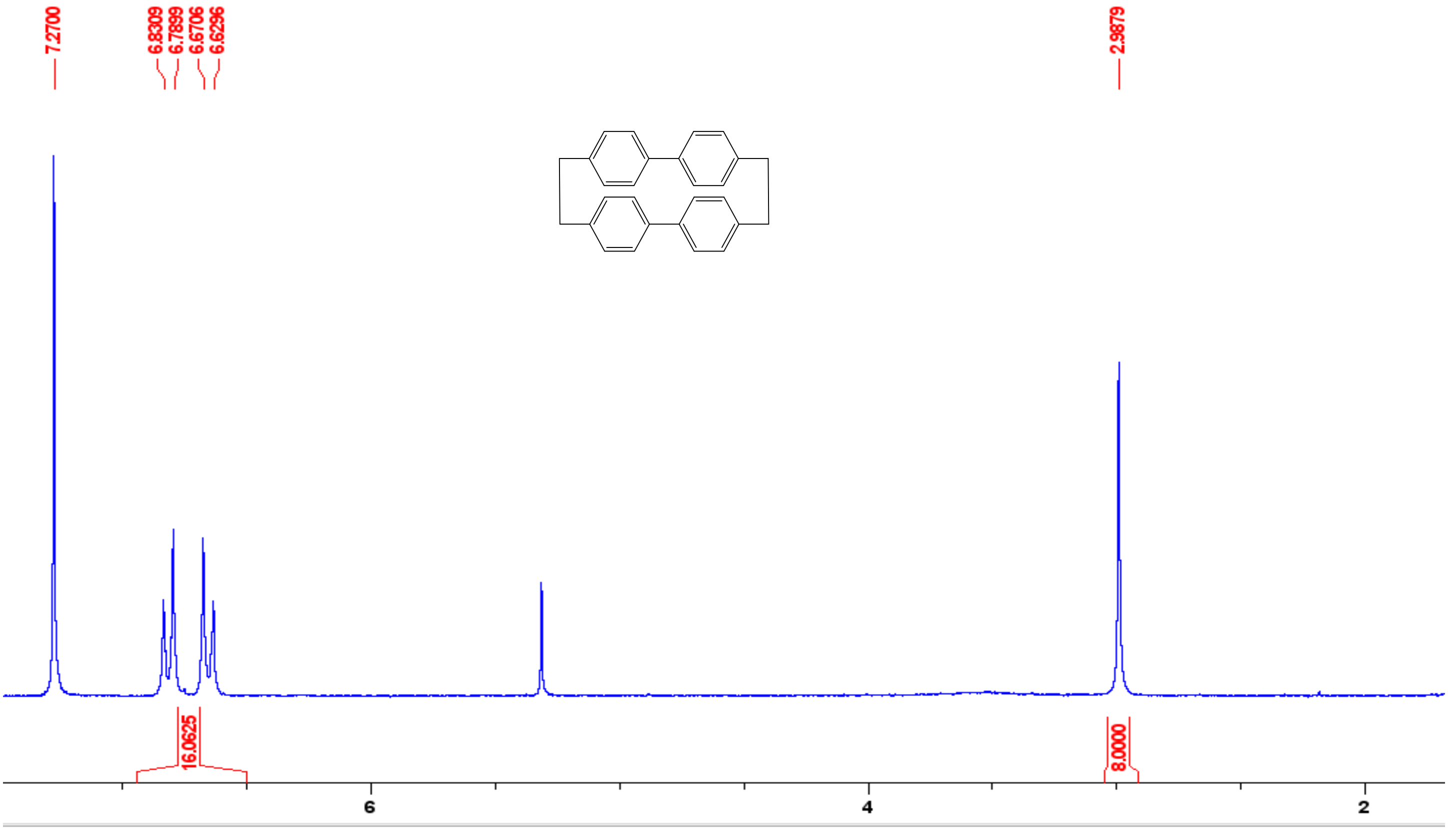

4) ^{13}C NMR

1,4-diphenyl-[2,2]paracyclophane

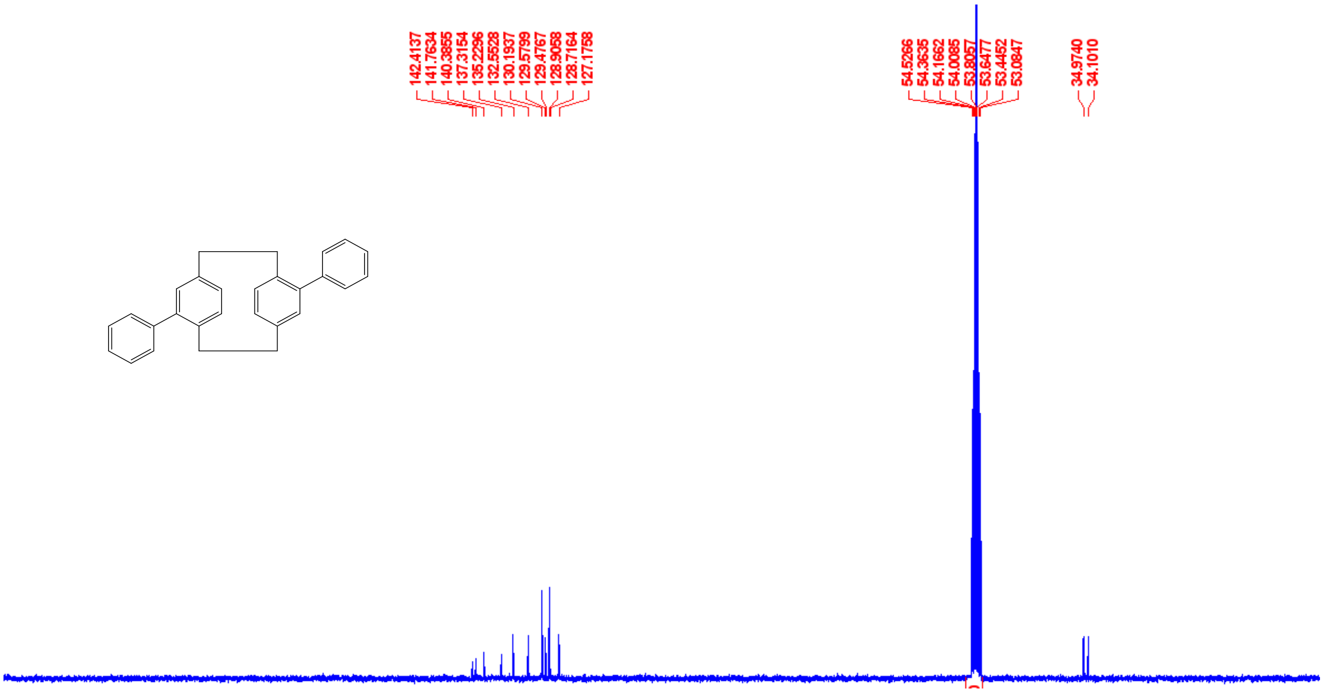

4,16-distyryl[2, 2]paracyclophane

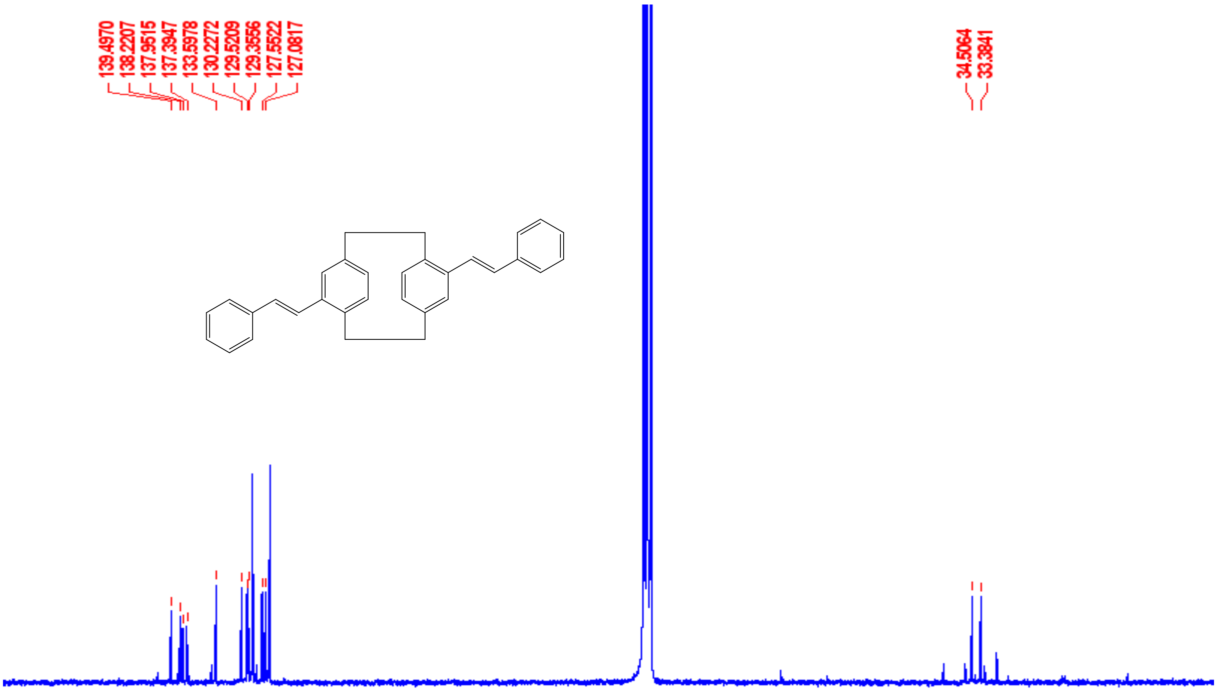

1,4-bis(bromomethyl)naphthalene

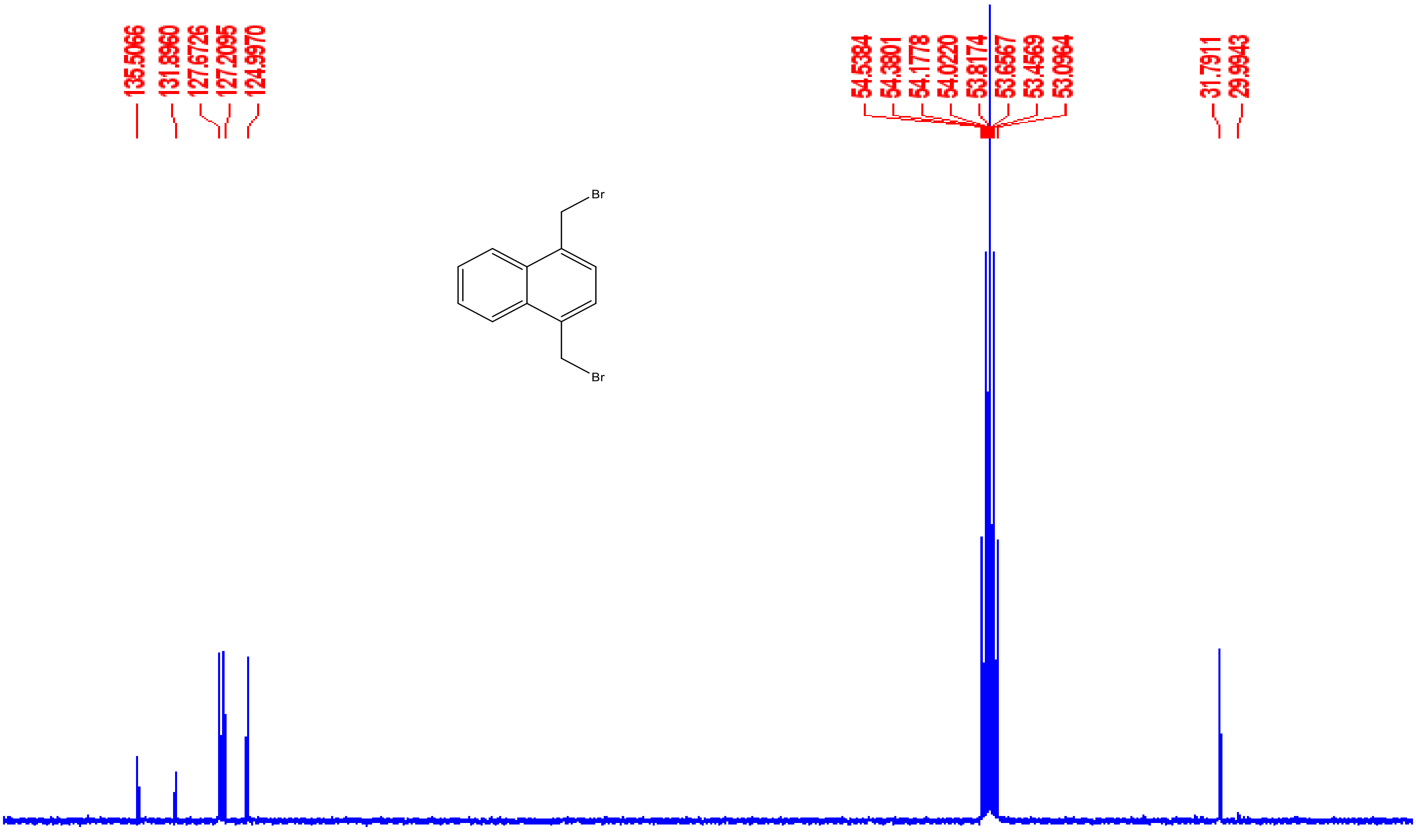

1,4(1,4)-dinaphthalenacyclohexaphane

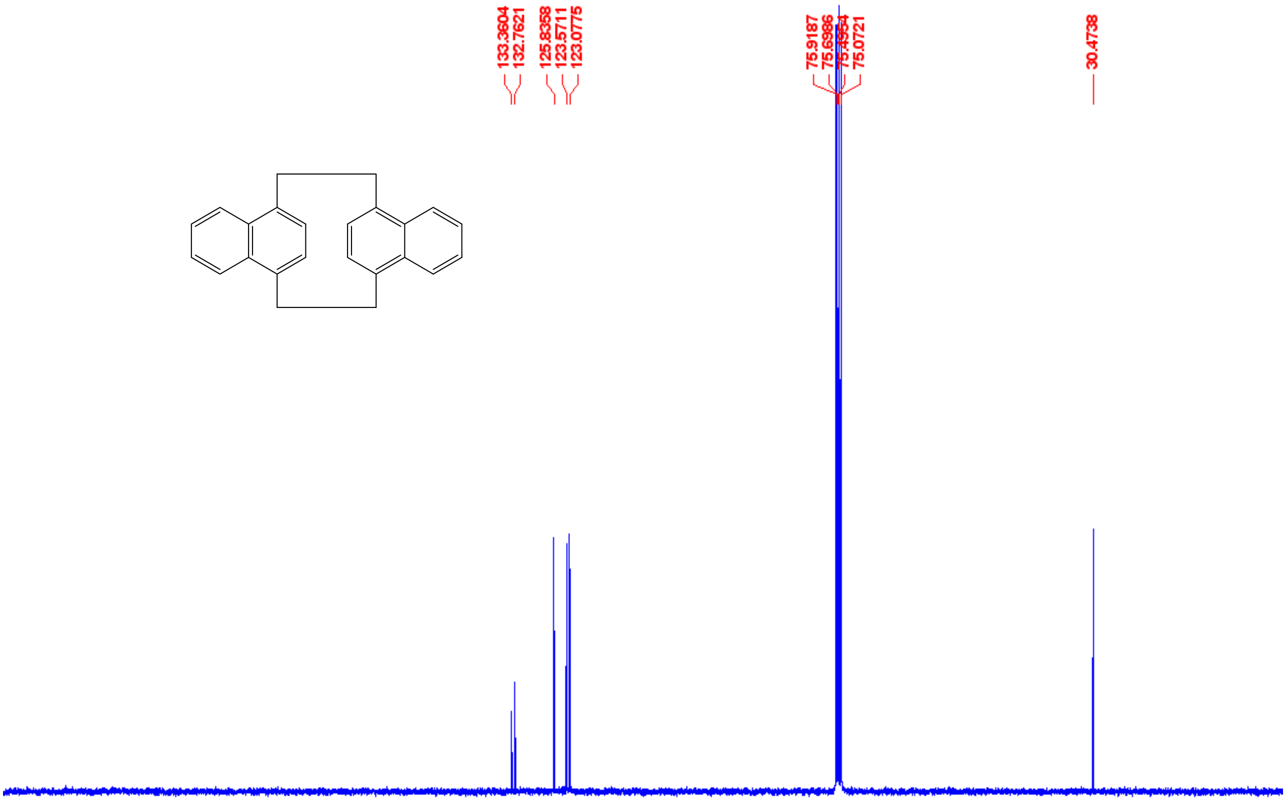

4,9-dithia-1,2,6,7(1,4)-tetrabenzenacyclodecaphane

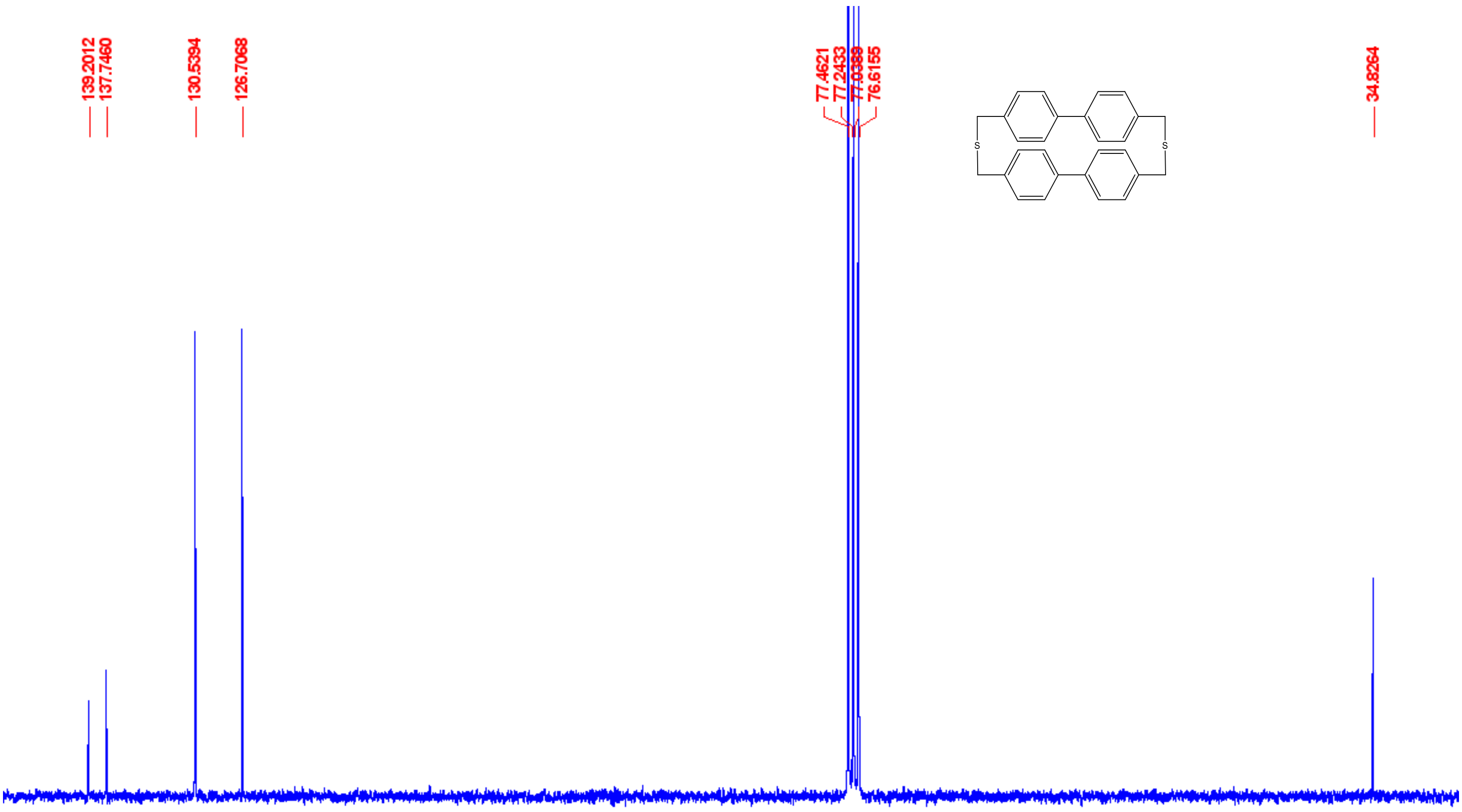

1,2,5,6(1,4)-tetrabenzena cyclooctaphane

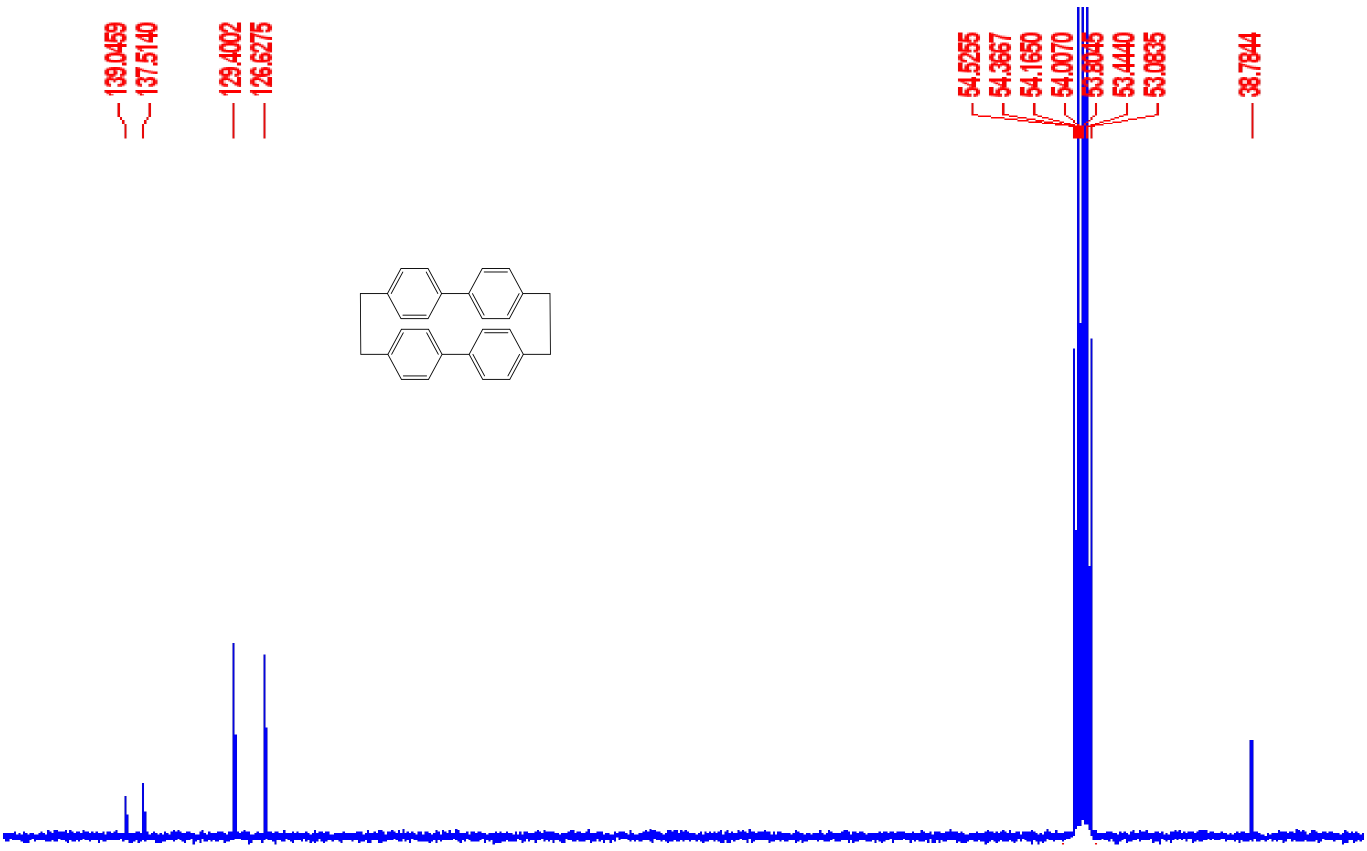

Supplement: Supplementary file 1 — Supporting Information [file OPEN-14-e202400207-s001.pdf]
